# A Clinicopathologic Study of Urinary Bladder Lesions Amongst North Indian Population: An Experience From a Tertiary Care Centre

**DOI:** 10.7759/cureus.59792

**Published:** 2024-05-07

**Authors:** Savita Agarwal, Pinki Pandey, Megha Ralli, Roopak Agarwal, Alka Yadav, Neetu Dwivedi

**Affiliations:** 1 Pathology, Uttar Pradesh University of Medical Sciences, Saifai, IND; 2 Pathology, Post Graduate Institute of Child Health, New Delhi, IND

**Keywords:** urinary bladder, turbt, smoking, radical cystectomy, infiltrating urothelial carcinoma, hematuria

## Abstract

Background: Urinary bladder cancer (UBC) is amongst the most common urological malignancies.

Aim: To study different types of urinary bladder lesions in the north Indian population and to correlate various clinical and pathological findings.

Materials and methods: The present prospective study was conducted on 100 cases undergoing transurethral resection of bladder tumor (TURBT) and/or radical cystectomy over a period of 2.5 years followed by histopathological examination. Liquid-based cytology for malignant cells in urine was also performed. Immunohistochemistry was employed for tumor typing wherever needed.

Results: A total of 100 cases were studied. Male to female ratio was 15.7:1 and most of the patients were in the sixth decade (40%). Painless hematuria was the commonest clinical presentation (60%) and smoking was the commonest risk factor (80%). The most common lesion was infiltrating urothelial carcinoma seen in 72 cases followed by papillary urothelial neoplasm of low malignant potential (PUNLMP) seen in eight cases. Grade and depth of invasion were assessed and correlated. Several variants of infiltrating urothelial carcinoma such as squamous differentiation, glandular differentiation, microcystic, clear cell, nested, and micropapillary were also identified. Clinical, cystoscopic and histopathological findings were correlated in all the cases.

Conclusion: Infiltrating urothelial carcinoma high grade was the most common bladder lesion identified and muscle invasion was more common with higher-grade lesions. A decade-younger age group was found to be more affected in the present series. Urine cytology for malignant cells is useful for early diagnosis of cancer. Immunohistochemistry is an important ancillary adjunct.

## Introduction

Urinary bladder cancer (UBC) is a common urological malignancy, the incidence of which is 350,000 per year worldwide. According to GLOBOCAN 2012, in the United States, the reported number of new cases of UBC was 74,690 and mortality was 4.4% [1].

As per the Indian cancer registry, UBC constitutes the ninth most common malignancy and accounts for an overall 3.9% of all cancer cases [2]. Urothelial cancers account for 5.6% of all male and 1.8% of female cancers in India, with an actual crude rate incidence of about one in 174 men and one in 561 women. Men are affected more than women. Women with UBC are more often diagnosed with a higher tumor stage than men [3]. Most cases of urothelial carcinoma of the bladder are present in patients over the age of 50 years but they can also occur in younger adults and children [4].

Hematuria is the most common presenting symptom [5]. High-grade cancers present predominantly above 60 years of age, while in <60 years of age, both high and low-grade cancers are common [6]. About 95% of bladder tumors are urothelial (transitional cell) type and are thus interchangeably called urothelial or transitional tumors, but squamous and glandular carcinomas can also occur [7].

For diagnosing bladder tumors, computed tomography urography (CTU) and MRI have an evident role. MRI has been evaluated for locoregional staging, including the evaluation of muscularis propria invasion. CTU offers a characterization of the upper urinary tract, with approximately 2-4% of patients with bladder cancer (BC) having concurrent upper tract urothelial carcinoma. Vesical Imaging-Reporting and Data System (VI-RADS) is an MRI scoring system developed in 2018 to standardize the imaging and reporting of bladder carcinoma on MRI. VI-RADS incorporates tumor appearance on T2-weighted imaging (T2W), diffusion-weighted imaging (DWI), and dynamic contrast-enhanced (DCE) imaging to assess the risk of tumor invasion [8].

## Materials and methods

The present descriptive cross-sectional observation study was conducted in the Department of Pathology, in collaboration with the Department of Neurosurgery at Uttar Pradesh University of Medical Sciences, Saifai, India from January 2021 to June 2023. Cases of urinary bladder lesions diagnosed on transurethral resection of bladder tumor (TURBT) and radical cystectomy specimens during that period were included.

Autolyzed and inadequate biopsies, known cases of UBC already on treatment, patients with non-neoplastic lesions, inflammatory lesions of the urinary bladder such as acute and chronic cystitis, and patients not giving written consent to participate in the study were excluded from the study.

Urine cytology for malignant cells was also performed by liquid-based cytology (LBC) technique wherever possible. Histopathological diagnosis was made according to the World Health Organization (WHO)/International Society of Urological Pathology (ISUP) 2016 and pTNM staging was done. Immunohistochemistry was employed for tumor typing and to differentiate between muscularis mucosae and propria wherever needed.

For statistical analysis, the whole data was entered into a Microsoft Excel master sheet (Microsoft® Corp., Redmond, WA) and analyzed using Statistical Package for the Social Sciences (IBM SPSS Statistics for Windows, IBM Corp., Version 20.0, Armonk, NY). As the data was qualitative, the Chi‑square test was used to assess the association between these parameters. A value of P <0.05 was taken as significant and <0.01 as highly significant; whereas, P >0.05 was taken as non-significant.

## Results

A total of 100 cases were studied. Male to female ratio was 15.7:1 (Table 1). The age range of the patients was 26-80 years. The maximum number of patients was in the sixth decade accounting for 40 cases, followed by the seventh decade accounting for 26 cases. The least common age group affected was the second and third decades with four patients in each decade.

**Table 1:** Distribution of cases according to gender

Sex	Number of cases (%)
Male	94 (94%)
Female	06 (6%)
Total	100 (100%)

Painless hematuria alone was the most common clinical presentation followed by hematuria with other symptoms such as urinary retention, burning micturition, and loin pain as shown in Table 2.

**Table 2:** Clinical presentation of patients with bladder tumor

Clinical presentation	Number of cases (%)
Painless hematuria	60 (60%)
Hematuria with burning micturition	08 (8%)
Hematuria with urinary retention	16 (16%)
Urinary retention	08 (8%)
Burning micturition	06 (6%)
Loin pain	02 (2%)
Total	100 (100%)

Smoking was identified as the single commonest risk factor present in 80 cases either alone or in combination with other risk factors (Table 3).

**Table 3:** Number of cases with associated risk factors

History of risk factor exposure	Number of cases (%)
History of smoking alone	14 (14%)
Farmer (possible exposure to chemicals like fertilizers and pesticides)	12 (12%)
Industrial worker (aromatic amine)	04 (4%)
Farmer + smoker	60 (60%)
Industrial worker + smoker	06 (6%)
No identifiable risk factor	04 (4%)
Total	100 (100%)

On cystoscopy, the majority of the lesions involved the posterior wall of the urinary bladder as seen in 30% of cases and the least common location was involvement near the left ureteric orifice in only two cases (2%). The most common growth pattern observed was papillary mass seen in 76% of cases while lobated growth was least common (Table 4).

**Table 4:** Cystoscopic findings of bladder tumors

Location of growth							
Right antero-lateral wall	Left antero-lateral wall	Posterior wall	Dome	Entire cavity	Near the left ureteric orifice		Involving ≥ 2 locations
20 (20%)	18 (18%)	30 (30%)	4 (4%)	8 (8%)	2 (2%)		18 (18%)
Growth pattern and size range							
Papillary	Polypoidal	Ulcerated	Seaweed like	Fungating	Lobated	Red friable and heaped mucosa	
76 (76%)	06 (6%)	04 (4%)	04 (4%)	04 (4%)	02 (2%)	04 (4%)	
2-6cm	<3cm	1-4cm	1-2cm	1-4cm	2.5cm	2-5cm	

**Figure 1:**
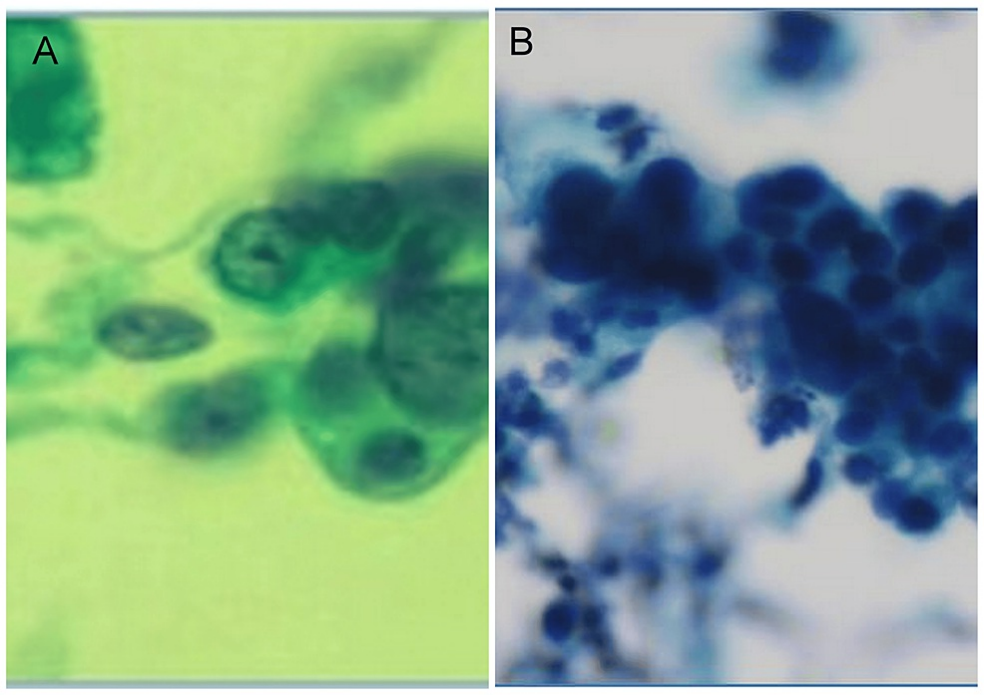
Urine cytology (A) Fragments of high-grade urothelial carcinoma with SurePath processing. The background is relatively free of inﬂammation and blood. (B) A group of malignant cells with irregular nuclear membranes and prominent nucleoli is seen (Giemsa; 400X).

Urine cytology for malignant cells was available in 32 out of 100 cases and 18 out of 32 cases were found to be positive for malignant cells on cytology (Figure 1).

**Figure 2:**
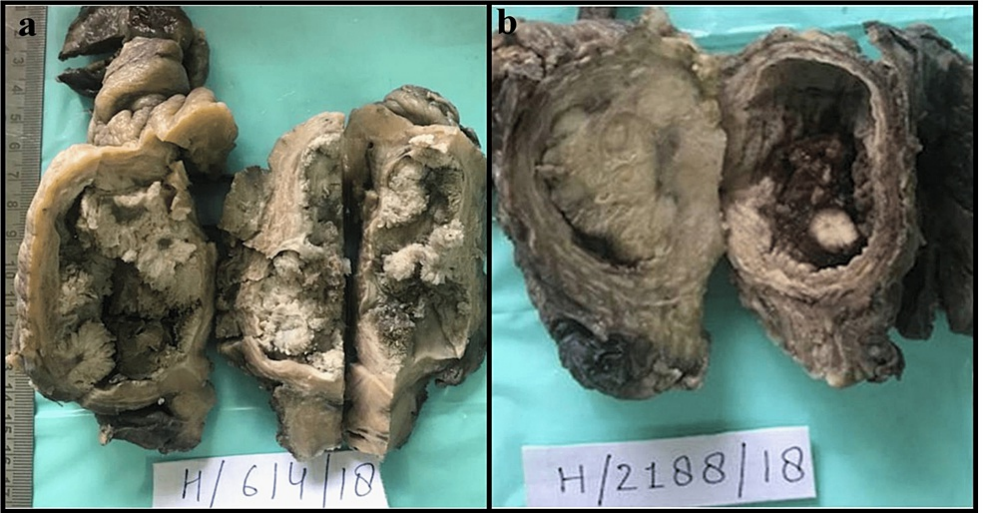
Radical cystectomy specimen: (a) infiltrating papillary urothelial carcinoma; (b) infiltrating urothelial carcinoma high grade.

Tables 5-6 show the specimen types and diagnoses rendered on histopathology respectively (Figure 2). The most common lesion was infiltrating urothelial carcinoma accounting for 72 cases (72%) out of which 58 cases (58%) and 14 cases (14%) were high and low grade respectively. The second most common lesion was papillary urothelial neoplasm of low malignant potential (PUNLMP) accounting for eight cases (8%). Five cases of squamous cell carcinoma (SCC) and one case of small cell neuroendocrine carcinoma were also seen.

**Table 5:** Types of specimens and their distributions TURBT: transurethral resection of bladder tumor

Types of specimens	Number of cases (%)
TURBT alone	80 (80%)
Radical cystectomy	06 (6%)
TURBT + radical cystectomy	14 (14%)
Total	100 (100%)

**Table 6:** Histological diagnosis of bladder tumors as per WHO/ISUP 2016 ISUP: International Society of Urological Pathology; PUNLMP: papillary urothelial neoplasm of low malignant potential

Histological diagnosis		Number of cases (%)
Papilloma		02 (2%)
Inverted papilloma		02 (2%)
PUNLMP		08 (8%)
Noninvasive papillary urothelial carcinoma low-grade		06 (6%)
Noninvasive papillary urothelial carcinoma high-grade		04 (4%)
Infiltrating urothelial carcinoma	Low grade	14 (14%)
	High grade	58 (58%)
Squamous cell carcinoma		05 (5%)
Small cell neuroendocrine carcinoma		01 (1%)
Total		100 (100%)

**Figure 3:**
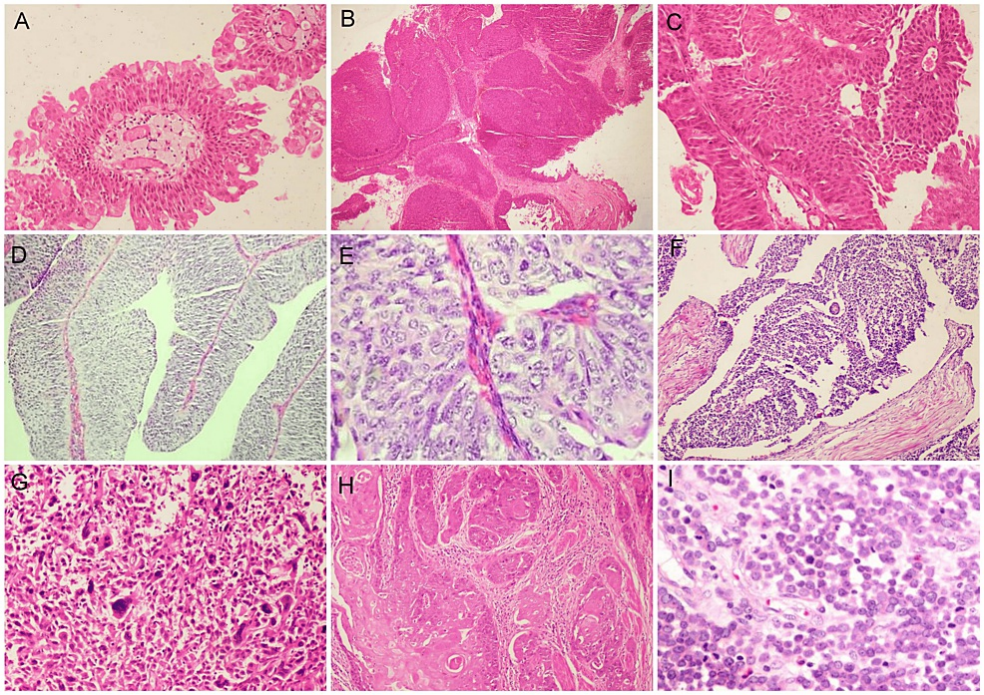
Histological diagnosis of bladder tumors as per WHO/ISUP 2016 (A) Papilloma. Lined by benign urothelial cells with central fibrovascular core (H&E 200X); (B) Inverted papilloma. Inward bulbous proliferation of urothelial cells (H&E 40X); (C) Papillary urothelial neoplasm of low malignant potential. Focal fusion of papillae with preserved nuclear polarity (H&E 200X); (D) Non-invasive urothelial carcinoma - low grade (H&E 200X); (E) Non-invasive urothelial carcinoma - high grade (H&E 400X); (F) Infiltrating urothelial carcinoma with invasion into muscularis propria (H&E 100X); (G) High-grade infiltrating urothelial carcinoma showing marked nuclear pleomorphism and multinucleation (H&E 400X); (H) Pure squamous cell carcinoma of urinary bladder (H&E 100x); (I) Small cell neuroendocrine carcinoma (H&E 200X). ISUP: International Society of Urological Pathology

Benign lesions like papilloma and inverted papilloma were uncommonly seen in two cases (2%) each (Figure 3). In cases of grade heterogenicity, grading was done according to the highest grade present.

**Figure 4:**
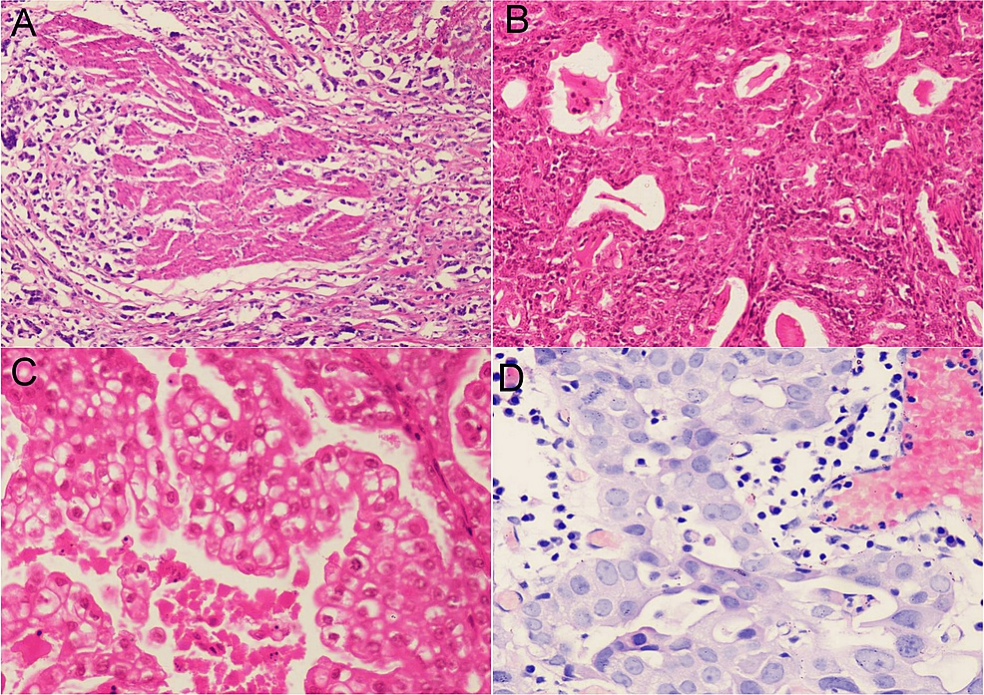
Infiltrating urothelial carcinoma and its variants. (A) Micropapillary variant of infiltrating urothelial carcinoma (H&E 100X); (B) Microcystic variant of infiltrating urothelial carcinoma (H&E 200); (C) Clear cell variant of infiltrating urothelial carcinoma (H&E 400X); (D) Infiltrating urothelial carcinoma with glandular differentiation (H&E 400X).

Several variants of infiltrating urothelial carcinoma were also recognized such as micropapillary accounting for six cases (8.33%) out of 72 cases, infiltrating urothelial carcinoma with squamous differentiation in four cases (5.56%) and two cases (2.78%) each of microcystic, nested, clear cell and infiltrating urothelial carcinoma with glandular differentiation (Table 7) (Figure 4).

**Table 7:** Infiltrating urothelial carcinoma and its variants TURBT: transurethral resection of bladder tumor

Infiltrating urothelial carcinoma and its variants	Specimen type	Number of cases (%)
Infiltrating urothelial carcinoma	Radical Cystectomy-10	54 (75%)
	TURBT-44	
With squamous differentiation	Radical Cystectomy-02	04 (5.56%)
	TURBT-02	
With glandular differentiation	TURBT	02 (2.78%)
Microcystic	TURBT	02 (2.78%)
Nested	Radical Cystectomy	02 (2.78%)
Micropapillary	Radical Cystectomy-02	06 (8.33%)
	TURBT-04	
Clear cell	TURBT	02 (2.78%)
Total	Radical Cystectomy-16	72 (100%)
	TURBT-56	

**Figure 5:**
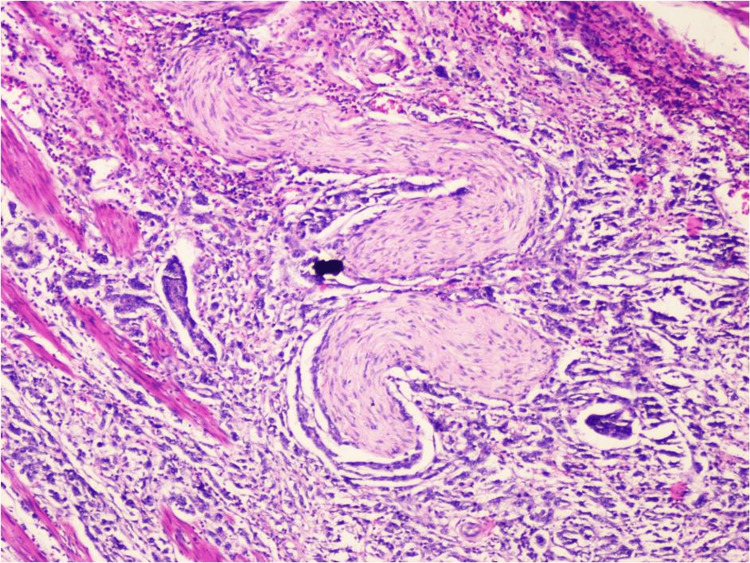
Perineural invasion in a case of infiltrating urothelial carcinoma (H&E 100X)

Infiltrating urothelial carcinoma (and its variants) was diagnosed in 56 TURBT and 16 radical cystectomy specimens. Tumor necrosis was present in 18 cases; perineural invasion was present in 14 cases (Figure 5) and focal granulomatous reaction with giant cells was seen in one case.

Out of 72 infiltrative urothelial carcinoma cases, lamina propria invasion was seen in 30 cases (41.67%), and of these 30 cases, muscularis propria was not present in 12 cases for assessment. Muscularis propria infiltration was present in the remaining 42 cases (58.33%) (Table 8).

**Table 8:** Cases of infiltrating urothelial carcinoma and depth of invasion

Depth of invasion	Number of cases of infiltrating urothelial carcinoma (%)
Lamina propria	30 (41.67%)
Muscularis propria invasion	42 (58.33%)
Total	72 (100%)

Muscle invasion was present in one case (2.8%) of infiltrating urothelial carcinoma low grade out of 14 cases and 41 cases (97.2%) of infiltrating urothelial carcinoma high grade out of 58 cases (Table 9).

**Table 9:** Muscularis propria involvement in cases of infiltrating urothelial carcinoma

Grade	Muscle involvement
Infiltrating urothelial carcinoma low grade (14 cases)	01 (2.8%)
Infiltrating urothelial carcinoma high grade (58 cases)	41 (97.2%)
Total	42 (100%)

**Figure 6:**
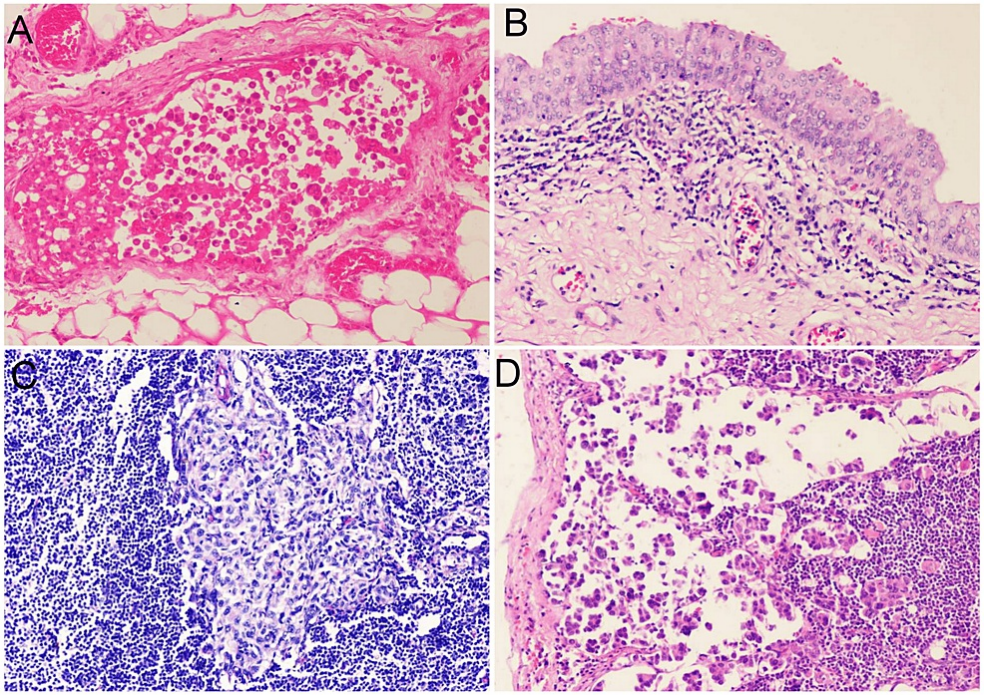
(A) Lymphovascular invasion in a case of infiltrating urothelial carcinoma (H&E 200X); (B) Urothelial dysplasia; (C) Urothelial carcinoma metastatic to pelvic lymph node (H&E 200X); (D) Micropapillary variant of infiltrating urothelial carcinoma metastatic to pelvic lymph node (H&E 200X).

Out of 20 cystectomy specimens, 16 cases were of infiltrating urothelial carcinoma. All cases were high-grade. Extension of tumor into perivesical fat was present in four cases, prostatic stroma was infiltrated in two cases where the resected margin was also involved by the tumor. Muscularis propria invasion was seen in all 20 cases. Lymphovascular invasion, urothelial dysplasia and lymph node involvement were present in 10 out of 16 cases each (Figure 6).

In four cases of radical cystectomy, a diagnosis of well-differentiated SCC was made. Both these cases showed either metaplastic or dysplastic squamous epithelium. Muscularis propria was involved in both cases; lymphovascular invasion and tumor extension into the seminal vesicle and prostate were present in two cases. Lymph node involvement was not seen in any of the five cases of SCC.

A single case of small cell neuroendocrine carcinoma was also diagnosed on TURBT. This case showed invasion into muscularis propria, small cell morphology and brisk mitotic activity. There was positive immunostaining for neuron-specific enolase (NSE), epithelial membrane antigen (EMA), P63, and P16 and negative for Chromogranin, CD117, TTF1 (thyroid transcription factor).

On, TNM staging the maximum number of cases (12/20 cases) were in the pT2 stage, where eight and four cases were in sub-stage pT2a and pT2b respectively. Of the remaining cases, four each were in T3a and T4b (Table 10).

**Table 10:** Histopathologic findings of radical cystectomy specimens

S. No.	Diagnosis	Type/variant	Urothelial/squamous dysplasia	Lymphovascular invasion	Extent of invasion	Resected margins of prostate	TNM staging
1.	Infiltrating urothelial carcinoma high-grade	Micropapillary	Urothelial dysplasia	Present	Perivesical fat and prostatic stroma	Involved by tumor	pT_4a_N_2_M_0_
2.	Infiltrating papillary urothelial carcinoma high-grade	_		Present	Perivesical fat	Free from tumor	PT_3a_N_2_M_0_
3.	Infiltrating urothelial carcinoma high-grade	_	Urothelial dysplasia	Present	Perivesical fat	Free from tumor	pT_3a_N_0_M_0_
4.	Well-differentiated squamous cell carcinoma	_	Metaplastic squamous epithelium (diffuse)	_	Muscularis propria	Free from tumor	pT_2b_N_0_M_0_
5.	Infiltrating urothelial carcinoma high-grade	_		_	Muscularis propria	Free from tumor	pT_2b_N_0_M_0_
6.	Infiltrating urothelial carcinoma high-grade	_	Urothelial dysplasia	_	Muscularis propria	Free from tumor	pT_2b_N_0_M_0_
7.	Infiltrating urothelial carcinoma high-grade	_	Urothelial dysplasia	Present	Muscularis propria	Free from tumor	pT_2b_N_2_M_0_
8.	Well-differentiated squamous cell carcinoma	_	Metaplastic and dysplastic squamous epithelium (diffuse)	Present	Perivesical fat, seminal vesicle and prostate	Free from tumor	pT_4a_N_0_M_0_
9.	Infiltrating urothelial carcinoma high-grade	squamous		_	Muscularis propria	Free from tumor	pT_2a_ N_1_ M_0_
10.	Infiltrating urothelial carcinoma high-grade	Nested	Urothelial dysplasia	Present	Muscularis propria	Free from tumor	pT_2a_ N_1_M_0_

## Discussion

The diseases of the urinary bladder both non-neoplastic and neoplastic are quite common. Bladder tumors are the seventh most common tumor worldwide. Urothelial carcinoma is the most common type accounting for 90% of all primary tumors of the bladder [9].

In the present study, there was a marked predominance of male gender in UBC cases with M:F ratio being 15.7:1, and the same was observed by Matalka et al. [10], Goyal et al. [5] and Joshi et al. [11] where male to female ratio was 9:1, 5.25:1 and 3.66:1 respectively; however, the ratio in our study was significantly higher than these studies. The reason for the higher incidence of UBC in males could be attributed to environmental exposure to carcinogens and smoking habits, while less incidence of BC in females could be due to infrequent exposure to individual carcinogens, less incidence of smoking [12,13] differences in the enzymatic processing of involved substances and also the cellular, and physiological responses [14]. Another possible explanation could be sex steroid and their receptor expression. The androgen receptor (AR) has been detected in normal bladder epithelium of both males and females as well as in bladder tumors. The importance of the AR signaling pathway in the development and progression of UCB has been put forward through experiments involving AR antagonists, small interfering RNA against the AR, and androgen deprivation. In addition, another study suggests the involvement of estrogen receptor β (ERβ) in development of UBC [14]. In females, the risk of development of UBC is greater in postmenopausal women as compared to other age groups.

The most commonly affected age group in our study was 51-60 years accounting for 40 cases (40%), followed by 61-70 years accounting 26 for cases (26%). Compared to the study by Goyal et al. [5] and Vaidya et al. [15] where the cases predominantly (33% cases) occurred in the seventh decade thus slightly younger (sixth decade) age group was more commonly affected in our study.

In the present study, a total of 72 cases of infiltrating urothelial carcinoma were observed. In the age group of ≤ 60 years, 14 cases (19%) were low grade and 30 cases (42%) were high grade. In the age group of >60 years, only four cases (6%) were low grade and the remaining 24 cases (33%) were high grade. In the study of Joshi et al. [11], the number of cases of urothelial carcinoma was 51. In the age group of ≤60 years, the number of cases of high grade was 30 (29%) and low grade was 16 (16%). In the age group of >60 years, the number of cases of high grade was 40 (39%) and low grade was 16 (16%).

In our study, hematuria alone was the most common clinical presentation, followed by hematuria with other symptoms, the least common presentation was loin pain. Thus in our study cases presenting with hematuria with co-existing symptoms accounted for 84 cases (84%). This is correlated with the study of Ray et al. [16] where 240 cases (91%) of UBT presented with painless hematuria with co-existing symptoms.

The most common single risk factor for UBC identified in our study was smoking. Smoking alone was present in 14 cases (14%). While smoking along with other risk factors, such as exposure to agricultural and industrial chemicals like fertilizers, pesticides and aromatic amine was seen in 80 cases (80%). Similarly smoking was the most common risk factor in a study by Joshi et al. [10] where 44 patients (78%) had a history of smoking. The other reported risk factors are the presence of arsenic in drinking water and occupational exposure to 4,4-methylenebis (2-chloroaniline) (MBOCA) [17].

Urine cytology is well-accepted, non-invasive modality for the diagnosis of primary or recurrent UBC, by identifying abnormal urothelial cells in the voided urine or bladder wash. Urine cytology has high specificity (93-97%) and low sensitivity (30-92%). The sensitivity and specificity vary between low-grade and high-grade urothelial carcinoma [18].

Cystoscopically most of the lesions were present on the posterior wall seen in 30 cases (30%) followed by the right and left anterolateral wall. The most common growth pattern was papillary mass seen in 76 cases (76%) followed by polypoidal growth and other patterns as described. In the study of Rafique et al. [19] on cystoscopy most superficial tumors had papillary growth patterns and muscle-invasive tumors had solid configuration.

On histopathology, the most common lesions were infiltrating urothelial carcinoma accounting for 72 cases (72%) followed by PUNLMP 8%, non-invasive papillary urothelial carcinoma low grade 6%, non-invasive papillary urothelial carcinoma high grade 4% and well-differentiated SCC 5%, small cell neuroendocrine carcinoma 1%, papilloma 2% and inverted papilloma 2% (Table 6).

In our study, PUNLMP was seen in 16% of cases whereas it was reported in four cases (4%) by Goyal et al. [5] and 11 cases (10.28%) in study Vaidya et al. [15]. In our study cases of infiltrating urothelial carcinoma low grade accounted for only 14% of cases which was quite less, compared with the studies by Goyal et al. [5] and Vaidya et al. [15] where a number of cases were quite high accounting for 31 cases (31%) and 32 cases (29.91%) respectively.

In our study, total number of cases of infiltrating urothelial carcinoma high grade was 58 (58%), which nearly correlated with study of Goyal et al. [5] where the number of cases of infiltrating urothelial carcinoma high grade were 60.4%. However, the number of cases of infiltrating urothelial carcinoma high grade in our study was quite high when compared with the study of Vaidya et al. [15] where cases accounted for 32.7%.

Pure SCCs are less common and account for about 1.3% of bladder tumors in males and 3.4% in females. Tobacco smoking is an important risk factor for SCC of the bladder. In the present study, smoking was seen in 80 cases 80%. Schistosomiasis is another risk factor for BC. The association of *Schistosoma haematobium* infection with BC is well documented [20]. In our study, SCC accounted for 05% of all cancers which was slightly less than that reported by Joshi et al. [11] where it was seen in 7.14% (Table 6).

Infiltrating urothelial carcinoma has a predisposition for differentiation into several variants. These variants with their approximate prevalence as reported by WHO is as follows: squamous differentiation being the commonest (40%), glandular (18%), nested variant (rare), microcystic (rare), micropapillary variant (0.6-2.2%), lymphoepithelioma-like urothelial carcinoma and plasmacytoid variant (less than 10 cases have been reported). Sarcomatoid variant (0.6%), clear cell variant (fewer than 25 cases have been reported till date), lipid-rich variant (only 37 cases have been reported), undifferentiated carcinoma are also known [20].

In our study the most common variant of infiltrating urothelial carcinoma was micropapillary followed by infiltrating urothelial carcinoma with squamous differentiation, microcystic, nested, clear cell and infiltrating urothelial carcinoma with glandular differentiation, these findings were similar to that reported by Goyal et al. [5] where 5.61%, 1.12% and 1.12% of cases showed squamous differentiation, nested and glandular differentiation (Table 7).

The clinical significance of squamous and glandular variants is uncertain but it is assumed to be a bad prognostic feature. The nested variant is an aggressive neoplasm with a bad prognosis. Micropapillary is a variant of high-grade and high-stage urothelial cancers with a high rate of metastasis and morbidity [20].

In our study total number of infiltrating urothelial carcinoma with squamous differentiation were four (5.56%) out of 72 cases of infiltrating urothelial carcinoma. Squamous differentiation is the most common histological variation in urothelial carcinoma [21]. It is characterized by the presence of intercellular bridge or keratinization with any identifiable urothelial element. It has a high risk of recurrence and poor prognosis as an independent prognostic factor [22]. The primary differential diagnosis for this variant is pure SCC. The diagnosis of SCC is reserved for pure lesions without any associated urothelial component [20,23].

Glandular differentiation is very uncommon and may be present in about 18% of urothelial carcinomas of the bladder. The diagnosis of infiltrating urothelial carcinoma with glandular differentiation is made by presence of true glandular space within the tumor. These may be tubular or enteric with mucin secretion [24]. Differential diagnoses of glandular variant include adenocarcinoma of the bladder (both urachal and nonurachal), cystitis glandularis, cystitis cystica, von Brunn nests, microcystic and nested variants of urothelial carcinoma, and metastatic adenocarcinoma. The expression of CDX2 and C K20 in areas of glandular differentiation may be a useful marker [20,23].

The nested variant of infiltrating urothelial carcinoma (NVUC) is a newly described and rare urothelial carcinoma subtype. It is characterized by the presence of bland cytological features i.e. nuclei with little or no atypia. It behaves aggressively with invasion; metastasis and deaths are common. Diagnosis of nested variant is made by the presence of foci of anaplastic cells with enlarged nucleoli and coarse nuclear chromatin. This feature is most apparent in a deeper aspect of the tumor [25]. The differential diagnosis of NVUC includes von Brunn nests, cystitis glandularis, cystitis cystica, and nephrogenic adenoma [23].

Triki et al. [21] reported a case of a 33-year-old man with a large NVUC, with symptoms suspicious for bladder carcinoma and misdiagnosed as nephrogenic adenoma. Four years later, he underwent partial cystectomy for bladder enlargement and bulging lesions on ultrasonography and was diagnosed with infiltrating urothelial carcinoma low-grade involving lamina propria and muscularis mucosa. The tumor was composed of small nests and microcysts lined by a single layer of atypical flattened cells.

The microcystic variant is one of the rarest variants of invasive urothelial carcinoma. It is characterized by the formation of microcysts, macrocysts or tubular structures [26]. The differential diagnosis of microcystic variant is cystitis cystica and cystitis glandularis difficult to differentiate especially in small biopsy specimens. Microcystic variant expresses GATA3, SI OOP, CK7, CK20, p63, and high-molecular-weight cytokeratins (CKs), and to a lower extent uroplakin Ill and thrombomodulin [20,23].

The micropapillary variant is a rare but well-recognized variant of infiltrating urothelial carcinoma. Diagnosis of the micropapillary variant is made by the presence of micropapillary areas within conventional urothelial carcinomas. These are more aggressive tumors compared to conventional urothelial carcinomas. The differential diagnosis of micropapillary urothelial carcinoma includes metastatic micropapillary adenocarcinoma of the lung, breast, and ovary [23].

Clear cell urothelial carcinoma (CCUC) is a rare variant of urothelial carcinoma and its clinical significance has not been well elucidated. It is characterized by the presence of a clear cell pattern with glycogen-rich cytoplasm. Periodic acid-Schiff (PAS) and PAS-diastase (PAS-D) stains confirm that the clear cells contain glycogen. This pattern is important in differential diagnosis with clear cell adenocarcinoma of the urinary bladder and metastatic carcinoma from the kidney and prostate [27].

Immunohistochemically, urothelium cancers express various markers such as different types of keratin, carcinoembryonic antigen (CEA), cathepsin B (particularly in high-grade lesions), CA19-9, CD15, CD10, S100, GATA-3, etc. [28]. Uroplakins, specialized surface proteins found exclusively within urothelium, will stain the urothelial component but not the areas of squamous differentiation. Staining for the L1 antigen and CK 14 will highlight areas of squamous differentiation but not normal or neoplastic urothelial cells [23]. Urothelial carcinoma with glandular differentiation is CK7 positive and villin, CDX2, and CK20 negative while metastatic adenocarcinoma of the colon shows positive staining for villin, CDX2, and negative for CK20 and CK7 [23]. In the case of microcystic variant immunohistochemical study demonstrated strong immunoreactivity with CK7 and CK AE1/AE3 [23]. Micropapillary variants show variable keratin 7, keratin 20 and human epidermal growth factor receptor 2 (Her2) neu expression [29]. Immunohistochemically, CCUC is reactive for CK7 and GATA3 [23].

In our study lamina propria invasion in Infiltrating urothelial carcinoma was present in 30 cases (41.67%) and muscularis propria invasion was present in 42 cases (58.33%). In 12 out of 30 cases of infiltrating urothelial carcinoma muscularis propria was not identified on TURBT specimens.

Limitations of our study

It is a single-centre study and multicentre studies should be invited for a better understanding of the histopathology of urinary bladder neoplasm. Cystectomy specimens were not received for all cases undergoing a biopsy, so the final diagnosis was not confirmed. Long-term follow-up was not available.

## Conclusions

Infiltrating urothelial carcinoma (high grade) was the most common bladder lesion identified in the present study and muscle invasion was more common with higher grade lesions. A decade-younger age group was found to be more affected in the present series. Urine cytology for malignant cells is useful for early diagnosis of cancer. Immunohistochemistry is an important ancillary adjunct.

Rare cases like SCC and small cell carcinoma of the bladder are also known so immunohistochemistry should be done in doubtful cases to rule out rare lesions also. Clear cell and micropapillary variants should be differentiated from the neoplasm of the kidney, prostate and ovary.
